# Microbial Conversion of Toxic Resin Acids

**DOI:** 10.3390/molecules24224121

**Published:** 2019-11-14

**Authors:** Natalia A. Luchnikova, Kseniya M. Ivanova, Ekaterina V. Tarasova, Victoria V. Grishko, Irina B. Ivshina

**Affiliations:** 1 Institute of Ecology and Genetics of Microorganisms, Ural Branch of the Russian Academy of Sciences, 614081 Perm, Russia; luchnikova.n@mail.ru (N.A.L.); kseniya.cheremnikh@gmail.com (K.M.I.); ekaterina.tarasova.87@mail.ru (E.V.T.); 2 Department of Microbiology and Immunology, Perm State National Research University, 614990 Perm, Russia; 3 Institute of Technical Chemistry, Ural Branch of the Russian Academy of Sciences, 614013 Perm, Russia; grishvic@gmail.com

**Keywords:** diterpenoids, resin acids, biotransformation, biological activity

## Abstract

Organic wood extractives—resin acids—significantly contribute to an increase in the toxicity level of pulp and paper industry effluents. Entering open ecosystems, resin acids accumulate and have toxic effects on living organisms, which can lead to the ecological imbalance. Among the most effective methods applied to neutralize these ecotoxicants is enzymatic detoxification using microorganisms. A fundamental interest in the in-depth study of the oxidation mechanisms of resin acids and the search for their key biodegraders is increasing every year. Compounds from this group receive attention because of the need to develop highly effective procedures of resin acid removal from pulp and paper effluents and also the possibility to obtain their derivatives with pronounced pharmacological effects. Over the past fifteen years, this is the first report analyzing the data on distribution, the impacts on living organisms, and the microbial transformation of resin acids. Using the example of dehydroabietic acid—the dominant compound of resin acids in effluents—the review discusses the features of interactions between microorganisms and this pollutant and also highlights the pathways and main products of resin acid bioconversion.

## 1. Introduction

The pulp and paper industry is one of the largest industries in the world. According to the UN, pulp production has increased from 163.5 to 183.9 million tons since the beginning of the 21st century [1]. The rise in production capacity is accompanied by an increased pressure on ecosystems, mainly caused by industrial effluent discharge [2].

Produced by coniferous plants of *Pinaceae* Lindl, resin acids (RAs) are one of the dominant groups of toxic compounds in liquid waste of pulp and paper mills (PPMs) [3]. Because of the shortcomings of the existing treatment systems and high chemical stability of RAs, they accumulate (up to 1500 mg/L) in nearby water bodies. In natural environments, RA concentration decreases due to sorption on solids and bottom sedimentation, as well as due to accumulation in aquatic organisms [4]. Accumulated in tissues and organs of the aquatic biota representatives, RAs cause irreversible negative effects, like red blood cell hemolysis, hepatocellular damage, and ATP depletion of nerve cells [5,6]. RAs can have a strong toxic effect on humans, affecting epithelial cells, polymorphonuclear leukocytes, and gingival fibroblasts [7,8], providing in some cases a pronounced tumor-promoting effect [9] and genotoxicity [10,11].

In this context, it is essential to search for efficient means of ecotoxicant removal. Biotechnological methods of RA conversion are priority solutions in terms of efficiency, safety, and cost-effectiveness. Employing enzymatic activities of microorganisms allows treating these pollutants in one technological stage without the need to use expensive chemicals and under eco-friendly reaction conditions. Despite the obvious toxic effects on living organisms, RAs can be used as parent compounds to derive novel, pharmaceutically significant substances with a broad bioactivity spectrum [12,13].

This review summarizes the data on distribution, impacts on living organisms, neutralization of RAs, and production of compounds with pronounced pharmacological actions. It should be noted that, from the late 1990s [14,15] to the present time, no such work has been carried out. This is the first report analyzing findings on occurrence, effects on aquatic biota, and microbial transformation of RAs over the past fifteen years.

## 2. Distribution and Toxicity of RAs

**Figure 1:**
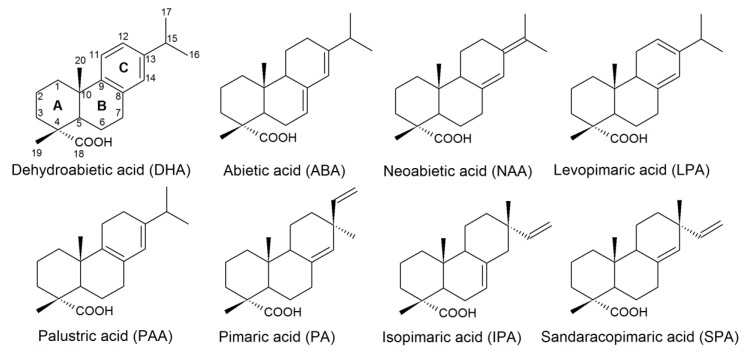
Structures of resin acids (RAs) found in pulp and paper mill (PPM) effluents.

RAs are diterpenic tricyclic monocarboxylic acids represented by two structural stereochemical groups—abietanes and pimaranes. The structures of abietane-type acids include an isopropyl group at the C-13 atom, whereas pimarane-type derivatives have two substituents—vinyl and methyl groups—at the same position [16]. The abietane acids found in PPM effluents contain abietic (ABA), dehydroabietic (DHA), neoabietic (NAA), levopimaric (LPA), palustric (PAA) acids, and pimarane-type compounds include isopimaric (IPA), pimaric (PA), and sandaracopimaric (SPA) acids (Figure 1).

RAs are major components of lipophilic extractives of generative and vegetative organs of conifers (pine, spruce, fir, and cedar) [17,18,19,20]. The RA content varies depending on the isolation source, season, and climatic conditions for growth of conifers. Thus, RAs and their derivatives are dominant components of fir and spruce seed extractives, being second to only di- and triglycerides [21]. At the same time, ABA dominates (up to 41%) in coniferous plant seeds from *Picea*, while PAA (up to 35%) is typical for *Abies* representatives [21].

The quantitative composition of extractives varies according to the object and the seasonal and climatic conditions for growth of coniferous plants [17]. A seasonal dynamics study of RA concentration in the wood of Scots pine (*Pinus sylvestris*) showed that their concentration increased from 3.17% (July) to 5.39% (January) of the total dry wood weight. The qualitative RA composition is also subject to seasonal variations. During the autumn-winter period, there was an accumulation of DHA from 2.00% (July) to 11.93% (November). A comparative analysis of pine wood samples at various geographical points (from 59° to 68° N) indicated that, towards northward, there was an increase in the total RA content with predominant PA. RAs are supposed to be involved in processes that favor pine adaptation to low temperatures. In addition, antibacterial [22], antifungal [23], and anti-inflammatory [24] properties of RAs protect the trees from pests and various pathogens [25].

The richest (up to 75% of total biomass) source of RAs—the galipot—is released from the damaged bark of conifers. The abundance of RAs in the galipot is variable and also depends on the nature of the source. For example, the Siberian cedar (*P. sibirica*) galipot is characterized by a relatively uniform content of several (five or more) RAs, while DHA is the dominant component of galipots in most other pine and spruce trees (Table 1) [3,26].

Woodworking and PPMs dealing with coniferous (soft) wood contribute to RA concentration (up to 1500 mg/L) in effluents and their subsequent presence in the environment, posing a toxic effect primarily on the aquatic fauna. The toxicity of most RAs has been studied using test organisms, like daphnia and fish. As seen from Table 2, the acute toxicity values depend on the solubility degree of RAs. In the row DHA ˃ ABA ˃ LPA ˃ NAA ˃ PA ˃ SPA ˃ IPA, solubility decreases from 5.11 to 1.70 mg/L, with IPA being the most toxic to the test organisms [14,27]. It should be noted that pH, temperature, and hardness of receiving waters affect the toxicity and solubility levels of RAs. Zanella [28] reported that when pH changed from 6.5 to 10.0, the acute toxicity level of DHA to daphnia and fish increased up to 76.9 and 45.5 mg/L, respectively.

Diluting and discharging into river and sea reservoirs is a widespread approach to PPM effluent disposal [29]. Analysis of filtered PPM effluents showed that only DHA and ABA—as the most soluble RAs—were detected in the water. At the same time, gas chromatography-mass spectrometry of the filtrate registered the presence of RAs, such as DHA, ABA, PA, and NAA, in the sediments [30]. With distance from the industrial effluent discharge sites, the total RA concentration decreased, while DHA concentration increased from 34% to 66% [29]. Thus, DHA is reported as highly resistant to abiotic environmental factors and can be used as an indicator of the open ecosystem pollution by PPM effluents. Undoubtedly, water systems contribute to reduced RA concentrations due to dilution. However, some authors believe that RAs (because of their lipophilic nature) are rapidly adsorbed on suspended solids and characterized by an increased settling capacity [29].

A decrease in RA concentration in water bodies is possible due to bioaccumulation of the latter in hydrobionts [4]. Concentration of RAs in fish can exceed the same value in water [31]. RAs were found in various organs and tissues of marine and river fish. The most significant accumulation was recorded in the blood plasma and liver of fish (Table 3). Studies of individual RAs revealed their different capabilities of accumulating in tissues of living organisms. Oikari et al. [31] noted that abietane acids accumulated in fish tissues to the greatest extent, while pimarane acids, in much smaller amounts. Probably, this pattern is associated with lower bioavailability and solubility of pimarane-type acids. Blood-accumulated RAs enter the liver and pose toxic effects to organisms. There are literature data relevant to RA effects on the erythrocyte and hepatocyte energetics, using the example of rainbow trout (*O. mykiss*). DHA introduction causes a sharp increase in oxygen consumption and heat release, leading to ATP depletion. In RA-contaminated fish, hepatitis is generally observed, resulting from the erythrocyte hemolysis and hepatocyte damage [6]. A similar effect has been reported for cells in the central nervous system of fish. For instance, the addition of DHA provokes increased oxygen consumption, a decreased ATP level, and promotes Ca^2+^ release from intracellular stores [5].

Widely spread in river and sea reservoirs, RAs are recorded (up to 8 µg/m^3^) as an inhalable particulate in the air at lumber mill work areas [33]. Analysis of smoke from the combustion of coniferous wood revealed DHA, ABA, PA, and IPA, as well as their hydroxy- and oxo-derivatives in suspended fine (2.5 µm) solids. At the same time, of all the substances (aliphatic and aromatic hydrocarbons, alcohols, acids, and phenols) detected, DHA was present in the greatest (23.8 µg/g of an extract) concentration [34].

There are data on RA migration from rosin, paper, and cardboard into food [35,36,37]. Currently, rosin consisting of more than 70% of RAs is used in dentistry as a component of periodontal dressings and cements, as well as a root canal filling [8]. Importantly, many RAs are resistant to environmental factors. For example, DHA has been preserved in pine bark compost for 12 months [38].

Ubiquitously distributed, RAs can influence both the aquatic fauna and humans. A mixture of individual RAs is characterized by high (concentration-dependent) cytotoxicity to human epithelial cells, polymorphonuclear leukocytes, and gingival fibroblasts [7,8]. Long-term exposure to RAs can provoke asthma and chronic pulmonary diseases in employees at wood-processing industries [39]. AA and DHA extracted from food packaging paper in high concentrations can promote a tumor formation [9]. Despite their obvious toxicity on the human body, some RAs can find potential applications in drug development, since they have anti-atherosclerotic [40], anti-inflammatory [24], antidiabetic [41,42], antitumor [43], osteoprotective [44], cytotoxic [45], antimicrobial [12], and anti-biofilm [46] actions.

## 3. Biodegradation of RAs

Biological treatment of PPM effluents is traditionally based on methods successfully employed in clean-up procedures of domestic wastewater. Biotreatments are classified into aerobic and anaerobic. Aerobic processes using aerated lagoons and activated sludge systems are the main options for complete degradation of organic material to CO_2_ and H_2_O or its transformation into eco-friendly compounds by natural microorganisms [47]. According to some authors [48,49], activated sludge and aerated lagoon treatments can reduce the level of abietane-type RAs in PPM effluents by 90%. However, removal of pimarane-type RAs is less efficient and does not exceed 60%.

It is generally accepted that most RAs can be removed by the aforementioned bioremediation systems. However, changes in the effluent composition, the properties of the degradation medium, the nutrient availability, and the state of the microbial community affect the treatment system efficiency and, in some cases, can lead to the release of more toxic and persistent compounds into the environment [14,50]. To prevent such disturbances, it is necessary to study in detail the composition of a microbial community and the role of each individual microbial species in biological treatment systems.

Because RAs are widespread in nature, microorganisms capable of degrading these ecotoxicants were discovered in various samples from river reservoirs [51], biological treatment systems [52,53], forest, agricultural, and Arctic soils [54,55], and soil and water contaminated with petroleum products [56]. Since the 1990s, a considerable number of pure bacterial and fungal cultures of RA degraders have been isolated (Table 4). The majority of aerobic bacterial isolates use RAs as a sole carbon source; still, there are data on bacteria that transform RAs but do not grow on them. Such bacterial cultures include proteobacteria isolated from compost by a group of Canadian scientists [57] and actinobacteria isolated from oil-contaminated soil and water bodies [51,56]. Fungal cultures usually transform RAs into hydroxy derivatives without using RAs as a source of carbon. The biodiversity of microbial biodegraders is basically represented by Alpha-, Beta-, Gamma-, and Deltaproteobacteria (Table 4). Data on the use of gram-positive bacteria for RA biodegradation are still scarce. The work has mainly been done employing actinobacteria and bacilli.

**Scheme 1:**
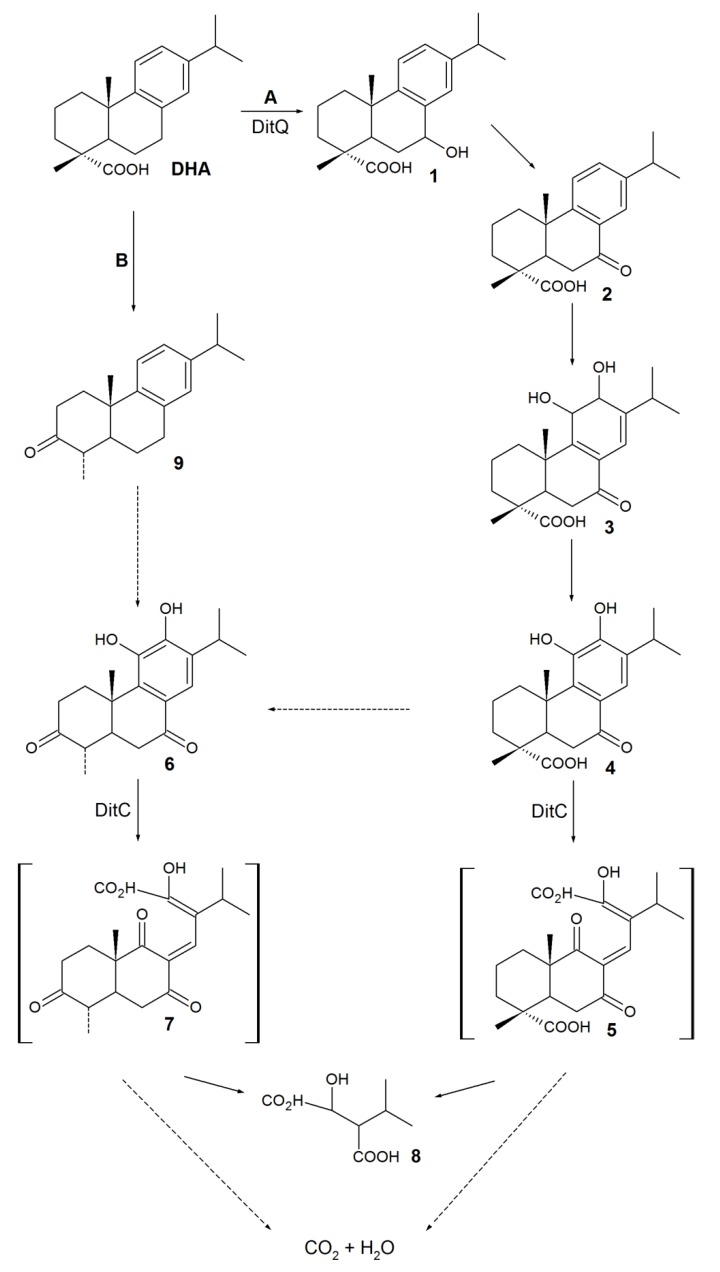
The proposed pathway for bacterial DHA degradation.

A general DHA biodegradation pathway was proposed based on genetic investigation of bacterial strains capable of degrading the abietane RAs isolated from a PPM (Scheme 1) [70,71,72].

**Scheme 2:**
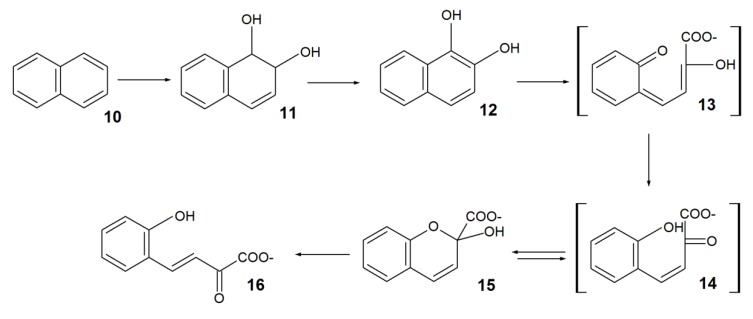
Bacterial degradation of polycyclic aromatic hydrocarbons (PAHs) on the example of naphthalene.

Presumably, the first step of biodegradation includes C-7 hydroxylationof DHA (Scheme 1, pathway A) or C-3 oxidation followed by decarboxylation to 3-oxo-dehydroabietin **9** (Scheme 1, pathway B). Compounds **1** and **9** were detected simaultaneously, which did not allow judging on the direction of aprimary oxidative reaction at C-3 and C-7 positions. In this regard, two alternative pathways for the intermediate **6** formation were suggested. The discovery of the DitA dioxygenase complex from *P. abietaniphila* BKME-9 [70] catalyzing the formation of 7-oxo-11,12-dihydroxy-8,13-abietadienic acid **3** from 7-oxo-derivative **2** gives evidence in favor of pathway A. Aromatization of diol **3** via 11,12-dehydration is supposed to lead to 7-oxo-11,12-diol **4**, and the latter is further oxidized and decarboxylated at C-3 to form an intermediate **6**. Identification of *P. abietaniphila* BKME-9 dioxygenase involved in the *meta*-cleavage of diterpenoids and detection of 2-isopropylmalic acid **8** entail that diols **4** and **6** could be further degraded via the aromatic ring cleavage resulting in the formation of possible intermediates **5** and **7** [15,71].The authors [15] believe that a general pathway of DHA biodegradation is somewhat similar to the initial pathways of bacterial degradation of polycyclic aromatic hydrocarbons (PAHs) (Scheme 2).

Using naphthalene **10** asan example, the authors [73] showed that a bacterial dioxygenase first catalyzed the formation of dihydrodiol **11** and then its aromatization, resulting in 1,2-dihydroxynaphthalene **12**. Subsequent cleavage of diol **12** occurred via the formation of unstable compounds **13**, **14**, and hemiacetal **15**. The latter, spontaneously or enzymatically, was isomerized to *trans*-*o*-hydroxybenzylidenepyruvate **16**. Assuming that DHA biodegradation proceeds similar to that of PAHs—via hemiacetal formation—it becomes clear why compounds **5** and **7** are not detected among biodegradation products [15].

Cheremnykh et al. [51] employed *R. rhodochrous* IEGM 107 and reported that metabolites of DHA were detected in the post-cultural medium when additional carbon and energy (*n*-hexadecane) sources were used. The mass spectra of metabolites corresponded to the known spectra of 7-oxo-DHA **2** and 7-oxo-11,12-dihydroxy-8,13-abietadienic acid **3** characteristic of *P. abietaniphila* BKME-9 [70]. The process of actinobacterial degradation of DHA by *R. rhodochrous* IEGM 107 cells seems to proceed likewise via C-7 oxidation followed by dihydroxylation and further *meta*-cleavage of the aromatic ring.

The formation of macroaggregates, the changes in morphometric parameters (an increased cell size) and the cell surface properties (an increased mean-square roughness, a decreased electrokinetic potential) of DHA-exposed actinobacteria were shown using different microscopic methods (namely, phase contrast, atomic force, and confocal laser scanning) and measurements of the cell electrokinetic potential [51]. The identified changes have been considered as mechanisms of actinobacterial adaptation to DHA exposure and, consequently, of their resistance to the DHA toxic effect.

**Scheme 3:**
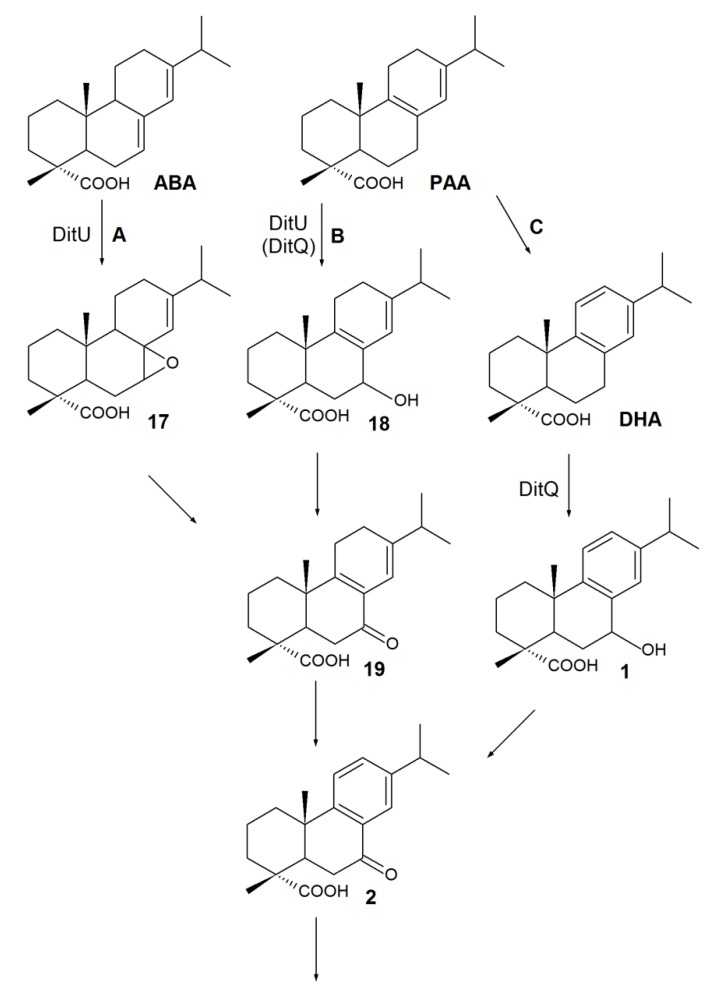
The proposed convergent pathway for abietane-type RA biodegradation.

Since the early 1990s, studies of the substrate specificities of RA-degrading bacteria have shown that the abilities of isolates to use DHA as the sole carbon source are in agreement with their ability to grow on any abietane-type derivatives [52,68]. The abietane-type RAs were previously thought to be metabolized via DHA [70]. However, using *P. abietaniphila* BKME-9 mutant strains, it was later shown that a mutation in gene *ditQ* limits pseudomonades’ growth in the presence of DHA but has little effect on the degradation of non-aromatic acids, like ABA and PA [60]. A genetic study of the biodegrading strains—*P. abietaniphila* BKME-9 [70,71] and *B. xenovorans* LB400 [62]—suggested a common convergent pathway for abietane-type RA biodegradation, resulting in the common intermediate7-oxo-DHA **2** (Scheme 3).

Presumably, the first stage of ABA biodegradation consists of oxidation to a 7,8-epoxy-ABA intermediate **17** (Scheme 3, pathway A), and the opening and rearrangement of an oxirane ring, leading to 7-oxo-PAA **19** formation [60,61,62,70,71]. The proposed mechanism (Scheme 3) explains the detection of compound **19** in the culture medium of *B. xenovorans* LB400 during ABA degradation [62]. Accumulation of 7-oxo-DHA **2** in the ABA degradation medium was probably due to aromatization of 7-oxo-PAA **19** to 7-oxo-DHA **2** [62]. Mineralization of PAA proceeded through two alternative pathways: via C-7 hydroxylation (compound **18**) followed by 7-oxo-derivate **19** formation (Scheme 3, pathway B) or through the key stage of aromatization to DHA (Scheme 3, pathway C) [60,61,62,70,71]. Pathway B is in agreement with the results reported by Smith et al. [62]. They detected the formation of 7-oxo-PAA**19** in the *B. xenovorans* LB400 culture medium supplemented with PAA. Thus, 7-oxo-PAA**19** is a “crossing point” of ABA and PAA metabolic pathways, while 7-oxo-DHA **2** is a “crossing point” of all abietane-type RAs.

The data on biodegradation pathways for pimarane-type RAs are sporadic. The aerobic gram-negative bacteria—known to grow on abietanes—cannot use pimarane acids as the sole carbon source. However, isolates growing on pimaranes can use both pimarane and abietane acids [52,67,68]. Present or absent in the molecules of these compounds, an isopropyl group is pivotal in dictating the RA biodegradation pathways. It was shown in [67] that isolates of *Pseudomonas* sp. IpA-1 and IpA-2, obtained from an enriched culture and grown in the presence of IPA, exhibited different degrading activities against abietanes. *Pseudomonas* sp. IpA-1 required IPA in the culture medium to effectively use abietanes, while the strain IpA-2 used abietanes as the sole carbon source.

**Scheme 4:**
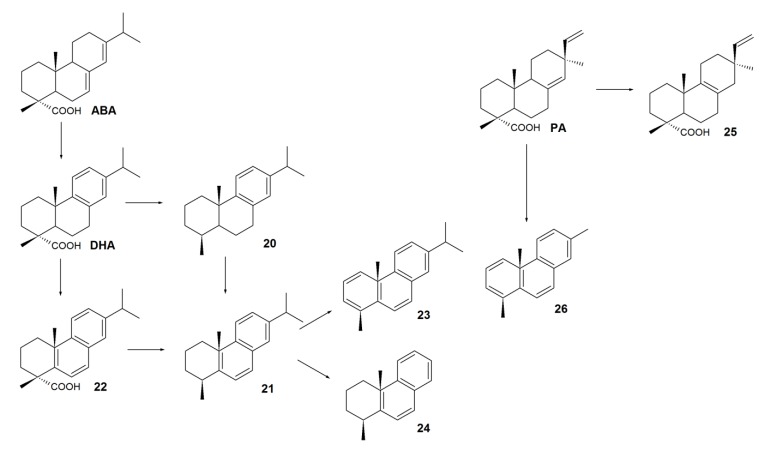
Proposed anaerobic pathways for ABA, DHA, and PA degradation.

The aforementioned degradation examples of various RAs have been mainly described for aerobic microorganisms. Under oxygen-free conditions, RAs could be biologically transformed; however, there is no convincing evidence of complete degradation of their carbon skeletons. Mohn et al. [54] showed that RAs are difficult to degrade under oxygen-free conditions. Moreover, pure anaerobic cultures capable of using RAs as the sole carbon source have not yet been isolated. Despite considerable difficulties associated with RA degradation under anoxic conditions, a group of New Zealand scientists led by Dr. Tavendale [74,75] carried out a large-scale study of DHA, ABA, and PA biotransformations under anoxic conditions into neutral derivatives and described for the first time new pathways of anoxic conversion of RAs in 1997. After 264 days of an anaerobic sediment incubation containing deuterium-labeled RAs, several compounds were isolated, suggesting a pathway for anaerobic metabolism of ABA, DHA, and PA (Scheme 4). 18-Norabietatrien **20** and tetrahydroretene **21** were registered as major products of anaerobic DHA degradation. Presumably, tetrahydroretene **21** was formed by the alternative pathways: either via ring B aromatization of 18-norabietatrien **20** or via carboxylation of abieta-5(10),6,8,11,13-pentaen-18-oic acid **22**. A small amount of tetrahydroretene **21** was transformed into retene **23** and 1-methyltetrahydrophenantrene **24**. Degradation of ABA under anaerobic conditions was also observed. However, the nature of this process is questionable (either biotic or abiotic) because the decreases in ABA concentration in the experiment and in the control were similar [74,75]. Degradation of pimarane-type RAs under anoxic conditions is still unclear A slight increase in pimar-8-en-18-oic acid **25** concentration was detected [15]. It is likely that anaerobic biocatalysis of pimarane acids proceeds similarly to that of abietane acids in a multi-stage process with the pimanthrene **26** formed [74,75]. Remarkably, the large-scale processes of anaerobic treatment are not widely applied for RA removal from effluents. Apart from being time-consuming, the employment of closed systems leads to concentrating of effluents. In this case, high concentrations of RAs inhibit the enzyme systems of anaerobic microorganisms [14,76].

## 4. Biotransformation of RAs to Bioactive Compounds

Up to date, RA-derived compounds with different pharmacological effects—anti-inflammatory [77], antimicrobial [12,78], fungicidal [12,22,23,79], anxiolytic [80], antiviral [23], antitumor [23,43,81], and anti-angiogenic [82]—have been described. In addition, RA derivatives can be used as intermediates in the synthesis of bioactive compounds [13,83] and pharmaceuticals [84,85]. To obtain novel compounds with biological properties, different techniques are used, particularly a chemical transformation [86,87]. Methods of chemical synthesis, however, often require expensive catalysts and introduction of protecting groups of reactive functional centers of the molecules. Widely known chemical conversions of RAs usually include classical transformations at rings B and C [83] because it is very difficult to perform regio- and stereoselective reactions at ring A using chemical methods. An alternative approach to structural modifications of natural compounds—particularly RAs—is microbial biotransformation, which does not require aggressive chemicals, proceeds in one technological stage, and is highly regio- and stereoselective [88,89]. Using inhibitors of enzyme systems allows for some intermediates of the microbial RA degradation with significant biological activities. For example, 7β-hydroxy-DHA **1** (Table 5)—a frequently registered product of DHA biotransformation—has antimicrobial, fungicidal, and selective antitumor properties [90,91]. Directed microbial transformations provide novel products unusual for the RA biodegradation pathways previously proposed.

Numerous abietane-type RA bioconversions using fungal strains have been reported. Basically, fungi modify a substrate molecule by stereoselective hydroxylation. Hydroxyl groups can be introduced at various positions of the molecule. In the case of RAs, hydroxylation reactions have been most frequently registered at C-1, C-2, C-7, C-15, and C-16 carbon atoms. DHA and ABA are usually used as substrates for directed transformation using fungi. Table 5 shows monohydroxy derivatives with biological activities obtained using fungal cultures.

**Figure 2:**
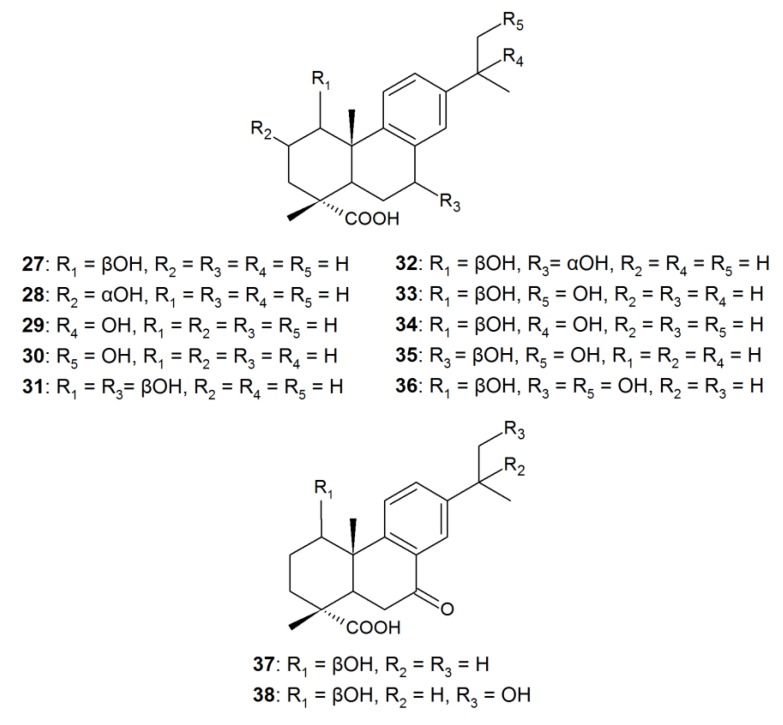
Structures of RA transformation products using fungi.

In addition, hydroxylation of DHA can proceed selectively or lead to the accumulation of several hydroxy derivatives of DHA. For example, when cultures of *C. elegans* TSY 0865, *R. stolonifer* ATCC-10404, *G. fujikuroi* ATCC-10704, and *C. aphidicola* IMI-68689 were used, three regioisomeric monohydroxy derivatives of DHA—acids **27**, **29**, and **30**—exhibiting an antibacterial effect and an inhibitory activity against α-glucosidase were the biotransformation products (Figure 2) [22]. Fungi are capable of transforming RAs into di- and trihydroxy derivatives at C-1, C-2, C-7, C-15, and C-16 positions. For example, in addition to 1β-hydroxy-DHA **27** and 7β-hydroxy-DHA **1**, a 1β,7β-dihydroxy derivative **31** with an antimicrobial activity was registered among the products of DHA biotransformation using *A. niger* cells [90]. In case of *P. gigantea*, together with 1β-hydroxy-DHA **27**, the hydroxy derivatives of DHA—1β,7α-dihydroxy **32**, di- (**33**–**35**), and tri- (**36**) hydroxy derivatives—were observed, including those with a β-orientation of the hydroxyl group at C-7 [93]. Similar di- and trihydroxy DHA derivatives (**33**–**36**) were detected in the transformation medium of *Trametes versicolor* [93]. In addition to DHA hydroxylation by *P. gigantea* and *T. versicolor* cells, transformation of this acid to 7-oxo derivatives—1β-hydroxy-7-oxo-DHA **37** and 1β,16-dihydroxy-7-oxo-DHA **38**—was observed (Figure 2) [93]. At the same time, examples of selective fungal transformation of pimarane-type RAs are few because PA and IPA are recalcitrant substrates for microorganisms.

Directed biotransformations of abietane-type RAs by bacterial cells to compounds unusual for the above described biodegradation pathways (Scheme 1, Scheme 3) are less studied. For example, 7-oxo-**2** and 5α-hydroxy-7-oxo-DHA **41** accumulated in the culture media for DHA transformations using *Burkholderia* sp., *Cupriavius* sp., and *Pseudomonas* sp. with a modified *ditA1* gene [95].

**Scheme 5:**
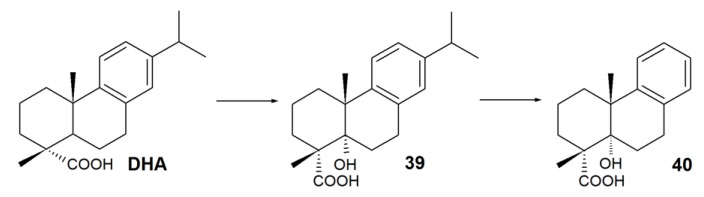
DHA biotransformation using *R. erythropolis* IEGM 267.

New metabolites—5α-hydroxy-DHA **39** and 15,16,17-trinor-abietane-type compound **40**—produced using *R. erythropolis* IEGM 267 cells pre-grown in the presence of DHA suggested a novel pathway of DHA biotransformation. Earlier, only single facts of hydroxylation at C-5 of the abietanes were described [95]. Biotransformation involving *R. erythropolis* IEGM 267 cells most likely occurs by oxidation of the parent compound molecule at C-5 of the carbon ring followed by deisopropylation of the aromatic ring (Scheme 5).

**Figure 3:**
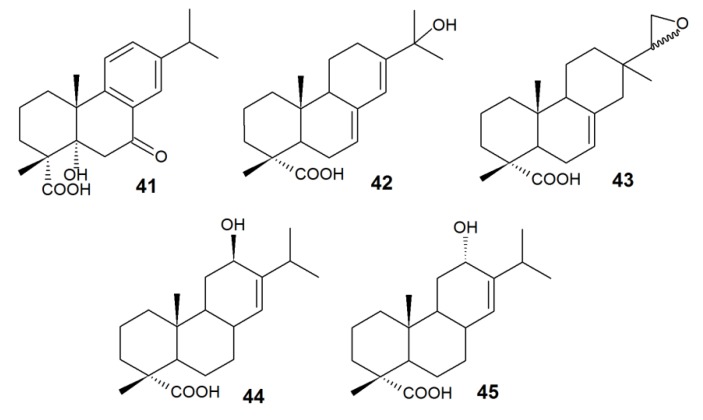
Structures of RA biotransformation products using bacteria and purified bacterial enzymatic complexes.

To degrade and transform RAs, enzymatic complexes as well as whole bacterial cells are often employed. The modified (by genetic transformation or gene cloning) cells of *Escherichia coli* with high enzyme efficiency are usually used to produce these complexes. Cytochrome-dependent bacterial enzymes CYP105A1 and CYP106A2 specific for RAs have been described in literature [25,96,97]. For example, CYP105A1 from *Streptomyces griseolus* catalyzed the formation of 15-hydroxy derivatives **29** and **42** from DHA and ABA, respectively, and 15,16-epoxy-IPA **43** from IPA [25]. 12-Hydroxy derivatives **44** and **45** were detected in the reaction medium containing ABA and CYP106A2 purified from *B. megaterium* cells (Figure 3) [96]. Enzymatic systems catalyze certain types of reactions, and this allows for the control of biotransformation processes. However, this method requires a significant investment of time and money as regards enzyme purification.

## 5. Conclusions

The most promising means of RA neutralization is the application of methods employing the enzymatic activity of microorganisms. Among the described cultures capable of complete RA degradation, the most common are mycelial fungi and proteobacteria isolated from RA-polluted sites. Gram-positive RA-degrading bacteria are, however, represented by only a few strains belonging to the genera *Bacillus*, *Mycobacterium*, and *Rhodococcus*. In recent years, intensive studies of RA biodegradation processes have favored the description of possible pathways for RA bioconversion.

Since the 2000s, transformation of RAs tailored to produce bioactive compounds for biotechnology needs has become increasingly important. So far, derivatives with significant anti-inflammatory, antimicrobial, fungicidal, anxiolytic, antiviral, antitumor, and anti-angiogenic activities have been described. It is of relevance due to the lack of therapeutic agents in certain areas of medicine (cardiovascular, tumor diseases, and immune system diseases).

It is noteworthy that, despite many processes of biological RA degradation and transformation having been described, the majority have significant drawbacks. Bacterial cultures usually exhibit their activities at RA concentrations not higher than 250 mg/L, while in effluents they can be exposed to RA concentrations exceeding this value (up to 1500 mg/L). The use of fungi capable of catalyzing a wide range of reactions implies certain risks due to their seed (spore) material and the ability to synthesize mycotoxins. To date, a number of actinobacterial strains related to *Dietzia maris*, *Gordonia rubripertincta*, *G. terrae*, *Rhodococcus erythropolis*, *R. rhodochrous*, and *R. ruber* and capable of converting higher (up to 500 mg/L) DHA concentrations have been discovered. The experimental data presented in this review create the prerequisites for the implementation of advanced technology solutions for an effective removal of RAs from PPM effluents.

## Figures and Tables

**Table 1:** RAs (%) in the galipot of conifers.

Acid	Scots Pine *P. silvestris*	Ordinary Spruce *P. excelsa*	Maritime Pine *P. pinaster*
Abietic	7.86	13.95	16.10
Dehydroabietic	64.58	50.08	23.50
Pimaric	10.86	7.57	10.80
Isopimaric	8.26	18.83	6.90
Unidentified	8.43	9.55	–

**Table 2:** Solubility and toxicity of RAs.

RA	Solubility, mg/L	Acute Toxicity (LD_50_), mg/L				
		Daphnia *Daphnia magna*, 48 h	Rainbow Trout *Oncorhynchus mykiss*, 96 h	Red Salmon *O. nerka*, 96 h	Silver Salmon *O. kisutch*, 96 h	Fathead Minnow *Pimephales promelas*, 96 h
DHA	5.11	1.28–6.35	0.77–1.32	0.50–2.10	0.75–1.85	2.10–3.20
ABA	2.75	0.68	0.72–1.53	0.20	0.40	2.38
LPA	2.54	0.50	0.61–1.00	–	–	–
NAA	2.31	0.35	0.63–0.71	–	–	1.30–1.70
PA	2.17	0.26	0.74–1.23	–	0.32	–
SPA	1.82	0.13	–	–	0.36	–
IPA	1.70	0.07	0.40–1.00	0.70	0.20	–

**Table 3:** RA bioaccumulation in fish organs.

Study Object	Concentration	Conditions	Reference
Fine flounder *Paralychthys adspersus* Small-eyes flounder *P. microps*			
Bile	30.5–41.9 µg/g, total RA content	Caught near the PPM effluent discharge site	[4]
Rainbow trout *O. mykiss*			
Bile	<200 µg/g DHA	After 57 days of exposure to PPM effluents	[32]
Blood plasma	155–318 µg/g DHA	After 4 days of exposure to DHA (1.2 mg/L) in water	[31]
Liver	98–103 µg/g DHA	After 4 days of exposure to DHA (1.2 mg/L)	
	202–351 µg/g, total RA content	After 2 days of exposure to a mixture of RAs (1.4 mg/L) in water	
Kidney	47–114 µg/g DHA	After 4 days of exposure to DHA (1.2 mg/L)	
	72–115 µg/g, total RA content	After 2 days of exposure to a mixture of RAs (1.4 mg/L) in water	

**Table 4:** Microorganisms capable of RA biodegradation.

Strain	Substrate	Reference
Gram-negative		
*Alcaligenes* sp. D11-13	DHA	[53]
*Betaproteobacterium* sp. DhA-71, DhA-73	DHA	[57]
*Burkholderia cepacia* F45L5	DHA, ABA, IPA	[58]
*Burkholderia* sp. DhA-54	DHA	[59]
*Burkholderia* sp. IpA-51	IPA	[59]
*B. xenovorans* LB400	DHA, ABA, PA	[60,61,62]
*Pseudomonas abietaniphila* BKME-9	DHA, ABA	[52,63]
*P. fluorescens* NRRL B21432	Mixture of RAs	[64]
*P. marginalis* E-001624	Mixture of RAs	[65]
*P. mohnii* IpA-2^T^, *P. moorei* RW10^T^	IPA	[66]
“Pseudomonas multiresinivorans” * (*P. nitroducent*) IpA-1 *	IPA	[67]
*P. reinekei* Mt1	IPA	[66]
*Pseudomonas* sp. A19-6a	ABA	[53]
*Pseudomonas* sp. DhA-92	DHA	[55]
*Pseudomonas* sp. IpA-2	IPA	[67]
*Pseudomonas* sp. IpA-93, IpA-95	IPA	[55]
*P. vancouverensis* Dha-51	DHA	[59]
*Ralstonia* sp. BKME-6	DHA	[52]
*Serratia marcescens* NRRL B21429	Mixture of RAs	[64]
*Sphingomonas* sp. DhA-33	DHA	[54,68]
*Sphingomonas* sp. DhA-95	DHA	[55]
*Xanthomonas campestris* NRRL B21430	Mixture of RAs	[64]
*Zoogloea ramigera* DhA-35	DHA	[68]
Gram-positive		
*Bacillus psychrophilus*	DHA	[69]
*Dietzia maris* IEGM 55^T^	DHA	[56]
*Gordonia rubripertincta* IEGM 104, IEGM 105, IEGM 109	DHA	[51]
*G. terrae* IEGM 150	DHA	[51]
*Mycobacterium* sp. DhA-55	DHA	[54]
*Mycobacterium* sp. IpA-13	IPA	[67]
*Rhodococcus erythropolis* IEGM 267	DHA	[51]
*R. rhodochrous* IEGM 107	DHA	[51]
*R. ruber* IEGM 80	DHA	[51]

Note: * The former name of the bacterial taxon is in upright font and in quotation marks. The current name of the taxon is given in parenthesis.

**Table 5:** Transformation products of abietane-type RAs using fungi, and their bioactivities.

Compound	Biological Activity	Biocatalyst	Reference
1β-hydroxy-DHA (**27**)	Antimicrobial, inhibitory activity against α-glucosidase	*Aspergillus niger*, *Cephalosporium aphidicola*, *Cunninghamella elegans*, *Fusarium moniliforme*, *F. oxysporum*, *Gibberella fujikuroi*, *Neurospora crassa*, *Phlebiopsis gigantea*, *Rhizopus stolonifera*	[22,90,91,92,93]
2α-hydroxy-DHA (**28**)	Antimicrobial, selective antitumor	*Mucor ramannianus*	[91]
7β-hydroxy-DHA (**1**)	Antimicrobial, fungicidal, antitumor	*A. niger*, *N. crassa*	[78,79,90,91,94]
15-hydroxy-DHA (**29**)	Anti-inflammatory. An intermediate of antiviral and antitumor agent synthesis	*C. aphidicola*, *C. elegans*, *G. fujkuroi*, *R. stolonifera*	[22,83]
16-hydroxy-DHA (**30**)	Antimicrobial	*C. aphidicola*, *C. elegans*, *G. fujkuroi*, *R. stolonifera*	[22]
